# Bacterial Diversity Associated with Terrestrial and Aquatic Snails

**DOI:** 10.3390/microorganisms13010008

**Published:** 2024-12-24

**Authors:** Konstantinos Apostolou, Canella Radea, Alexandra Meziti, Konstantinos Ar. Kormas

**Affiliations:** 1 Department of Ichthyology and Aquatic Environment, School of Agricultural Sciences, University of Thessaly, 384 46 Volos, Greece; apostolou@uth.gr; 2 Section of Ecology and Taxonomy, Department of Biology, National and Kapodistrian University of Athens, Panepistimioupolis Ilissia, 157 84 Athens, Greece; kradea@biol.uoa.gr; 3 Department of Marine Sciences, University of the Aegean, 811 00 Mytilene, Greece; a.meziti@aegean.gr

**Keywords:** gastropods, microbiota, symbiont, gut, freshwater, marine

## Abstract

The introduction of the holobiont concept has triggered scientific interest in depicting the structural and functional diversity of animal microbial symbionts, which has resulted in an unprecedented wealth of such cross-domain biological associations. The steadfast technological progress in nucleic acid-based approaches would cause one to expect that scientific works on the microbial symbionts of animals would be balanced at least for the farmed animals of human interest. For some animals, such as ruminants and a few farmed fish species of financial significance, the scientific wealth of the microbial worlds they host is immense and ever growing. The opposite happens for other animals, such as snails, in both the wild and farmed species. Snails are evolutionary old animals, with complex ecophysiological roles, living in rich microbial habitats such as soil and sediments or water. In order to create a stepping stone for future snail microbiome studies, in this literature review, we combined all the available knowledge to date, as documented in scientific papers, on any microbes associated with healthy and diseased terrestrial and aquatic snail species from natural and farmed populations. We conducted a Boolean search in Scopus, Web of Science, and ScienceDirect until June 2024, identifying 137 papers, of which 60 were used for original data on snail bacterial communities in the gastrointestinal tract, hepatopancreas, and feces. We provide a synthesis on how representative this knowledge is towards depicting the possible snail core microbiota, as well as the steps that need to be taken in the immediate future to increase the in-depth and targeted knowledge of the bacterial component in snail holobionts.

## 1. Introduction

Since the introduction of the holobiont concept [1], it has been established that all macroorganisms are structurally and functionally inseparable from their microorganisms, and they should be considered functioning physiologically and evolutionary as one biological entity [2]. These symbiotic microorganisms have multiple beneficial or harmful roles to their hosts, with the most pivotal being involved in the development, reproduction, behavior, nutrition, immunity, and stress response [3]. Their exact origin and mode of transmission remains, in most cases, rather elusive, at least when compared with their roles for their hosts’ lives.

Mollusca are an extraordinarily diverse phylum of invertebrates that radiated rapidly during the early Cambrian period (approximately 535–520 million years ago) [4]. Overall molluscan species-level diversity is estimated at 87,359 valid extant species and 41,899 accepted fossil-only species [5], and it is second only to the arthropods’ diversity [6]. Prior extremely high estimations of molluscan numbers based on expert opinions have critically been reviewed by [7], which listed species-level diversity from 70,000 to 76,000 extant valid species. The most diverse and disparate molluscan taxonomic classes are Gastropoda, Bivalvia, and Cephalopoda [4]. The class Gastropoda (gastropods: snails and slugs) occupies a broad range of niches in aquatic and terrestrial environments and includes 40,354 marine, 5368 freshwater, and 28,881 terrestrial accepted extant species [5]. Terrestrial gastropods form a highly varied group of mollusks, allocated in an extensive variety of ecosystems. Their vast majority are Stylommatophora pulmonates that dominate the terrestrial fauna in most regions of the world [8].

The species of terrestrial gastropods exhibit a remarkable diversity in their habitats and their life-history traits, influenced by many factors such as seasonality, climate, ecological tolerance, adaptation, and life stage of the parental individuals [9]. Climatic variations significantly influence terrestrial snails because these invertebrates are vulnerable to changes in temperature and water regime. Prolonged periods of drought combined with prevailing high temperatures impose considerable stress on them, affecting their metabolic responses [10,11]. To endure these conditions, terrestrial snails have evolved adaptive mechanisms that allow them to persist in hot and dry habitats [12]. Many terrestrial gastropods can be found across various vegetation types, including crops, vegetables, and fruit trees [13] and are considered as important crop pests. Some imported species have invaded natural habitats, with negative impacts on native biodiversity [14].

Terrestrial gastropods play a fundamental role in ecosystem by involving, directly or not, in decomposition processes nutrient cycling and soil formation [15,16,17]. Additionally, land snails serve as prey for various predators including birds, mammals, and insects [18]. Furthermore, terrestrial snails are recognized as bioindicators [19,20], providing valuable information about environmental conditions and biodiversity [21]. Despite their ecological importance, due to their minimal dispersal ability and dependency on specific microhabitat, land snails are highly vulnerable [18], and one-quarter (26%) of the species assessed by the International Union for Conservation of Nature (IUCN) specialists are under threat [22].

Marine gastropods inhabit all possible sea habitats such as estuaries, rocky and sandy shores, continental shelf, hydrothermal vents, abyssal plain, and sea trenches of the hadal zone [23,24,25,26]. Various ecophysiological adaptations have been evolved by gastropods to respond to the varying conditions of the aquatic environment. In marine gastropods, metabolic depression is observed during overwintering under extreme conditions [27] and proteomic changes are recorded in conditions of hypo-osmotic stress [28]. Even though marine gastropods represent only 2% of the mollusks fished worldwide [29], they have high commercial value since they are used as food, money, ornament, dye, and traditional Chinese remedies [30,31]. The neurotoxin peptides found in some predatory species are bioactive compounds of great biotechnological interest for applications in medicine [31]. Despite their diversity, as well as their ecological and economic importance, marine gastropods remain underrepresented in assessments listed by the IUCN and only a small portion (2.5%) of their species has been evaluated. Over one in ten (16%) of the marine gastropods assessed are considered to be at risk of extinction [22].

Freshwater gastropods are partially or wholly restricted to freshwaters and are found in nearly all continental permanent and temporary aquatic habitats such as rivers, lakes, streams, underground aquifers, springs, swamps, and ponds on every continent except Antarctica [6,32]. Freshwater pulmonate gastropods are mostly pond inhabitants which have lost their gills during their intermediate terrestrial phase and currently breathe air with a vascularized lung [33]. They can withstand lower oxygen tensions by surface breathing or by reliance on anaerobic metabolism [34] and survive in a wider range of extreme temperatures by better regulating their metabolic rate than most of the gill-bearing gastropods do [33]. Additionally, their eyes have fixed focal lengths adapted to vision both in water and air [35]. In extremely varying habitats, some freshwater gill-bearing gastropods can start their reproduction independently of their body size so that they increase the chance for maintaining a stable population size [36]. Freshwater pulmonate gastropods play a remarkable role as intermediate hosts in the transmission of many parasites of human and veterinary importance such as *Schistosoma mansoni* (Platyhelminthes: Trematoda), *Fasciola hepatica* (Platyhelminthes: Digenea), and *Angiostrongylus cantonensis* (Nematoda: Rhabditida) [37,38]. The transmission of parasites through gastropods is recognized as a major problem, resulting in human diseases with millions of cases worldwide and in the loss of productive capacity of livestock [37]. Over three in ten (33%) of the freshwater gastropods included in the IUCN Red List of Threatened Species are considered at risk of extinction [22].

Gastropods are significant components of marine and freshwater food webs and energy transfer pathways, being primary and secondary consumers as well as the prey of larger predators [39]. They are classified into carnivores, scavengers, herbivores, and omnivorous, according to their food type [4], and into predators, chemosynthetics, or photosynthetics, grazers, and suspension and deposit feeders on the basis of the feeding mechanisms they use [40,41,42,43,44].

Considering their aforementioned significant biological roles, there is no reason why, even today, snail microbiome research should remain so scarce, as with other animal species of both ecological and economic interest. Snails have a very complex gastrointestinal tract system (GIT), living on soil/sediment/water which have immense microbial diversity and abundance, and are of commercial interest both in wild and farmed populations (e.g., as novel diet formulations, etc.). On the other hand, the microbiota and microbiomes within their terrestrial and aquatic habitats could act as important background information on the snail’s adjacent microbial world. The current literature review aims to summarize the current knowledge on the bacterial diversity of snail-associated microorganisms.

## 2. Materials and Methods

**Figure 1:**
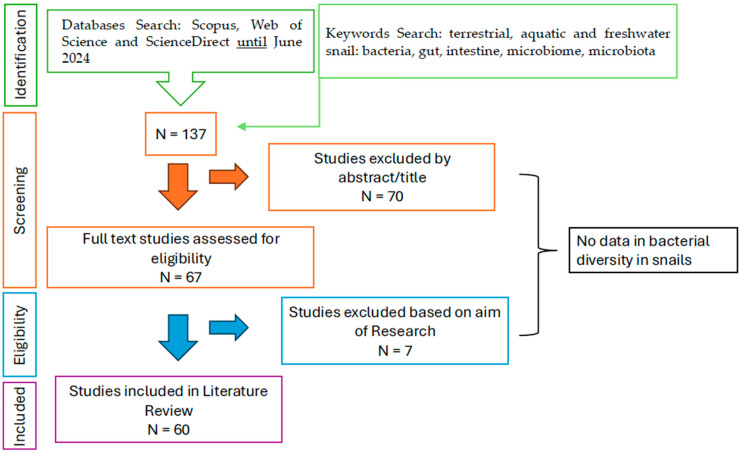
Flowchart of research and screening of the reviewed literature.

To date, there are few published studies dealing with the microbial symbionts of terrestrial, freshwater, and marine snails. In order to gather all papers for comparison across all the sources, we searched through databases for journals, articles, and book chapters of the peer-reviewed literature. Our objective was to combine all the available knowledge to date, as documented in published scientific papers only, on any microbes associated with healthy and/or diseased terrestrial and aquatic snail species from natural and farmed populations. The main objective of this review was to record the diversity breadth of bacterial taxa associated with species of snails across various habitats. Another goal was to identify the bacterial core of snails in each habitat (marine, freshwater, terrestrial). The bacterial core taxa in this review refers to a consistent set of bacterial species found in snails across all studies associated with a specific habitat. We performed a Boolean search in Scopus, Web of Science, and ScienceDirect until June 2024 in order to collect and compare every paper containing any of the following keywords: terrestrial, marine, freshwater, snail, bacteria, gut, intestine, microbiome, microbiota. The immediate outcome led to 137 papers, which can be found in the Supplementary Materials (List of papers used for this review S1). Of these, after being individually examined, 60 of them were used as only these provided original results on the snail microbial communities associated with the gastrointestinal tract, hepatopancreas, and feces. All these papers included bacterial communities only (Figure 1). The papers covered different snail species (terrestrial, freshwater, and marine) from all continents but Antarctica. In this review, we present the bacterial phyla of snails, the species of gastropods, the sampling sites where the snails were collected for investigation, and the methods used (culture dependent or independent).

▪ Graphic illustrations were performed using Circos software vo.63-10 (https://mk.bcgsc.ca/tableviewer/) (accessed on 30 July 2024) [45] and SankeyMATIC (https://sankeymatic.com/build/) (accessed on 16 August 2024) [46]. Also, ggplot 2 (v 3.5.1), UpSetR (v. 1.4.0), and ggupset (v. 0.4.0) packages in R Studio platform Version 2024.04.2+764 [47] with R version 4.4.1 were used for the visualization of results.

## 3. Results and Discussion

The studies analyzed included snails from terrestrial as well as marine and freshwater habitats. Also, the dataset analyzed included results from the enrichment and cultivation methods, as well as microbe identification using molecular techniques mostly by the diversity of the 16S rRNA gene. Results are presented in the increasing number of relevant papers found, starting from marine, then moving on to freshwater and, finally, terrestrial habitats. Finally, the comparison of all habitats is presented, as well as the occupancy between snails and their bacterial communities.

**Figure 2:**
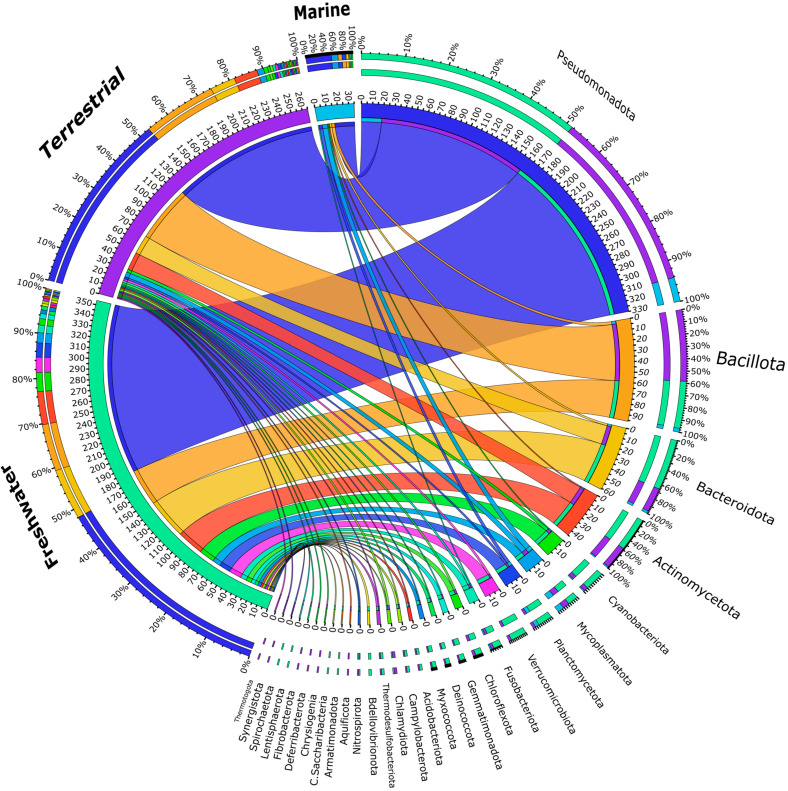
Circos plot of the corresponding relative abundance between snails’ habitat and bacterial phyla.

A total of 33 snail species from various habitats (land, marine, freshwater) will be examined in this literature review. There are 16 freshwater snail species, 10 terrestrial, and 7 marine species. The most abundant phylum regardless of environment, was Pseudomonadota, followed by Bacillota and Bacteroidota (Figure 2). The researchers in [48] reported similar observations in freshwater snails. Pseudomonadota represent 48% of the bacteria in freshwater snails, 53% in terrestrial, and 54% in marine (Figure 2), based on the studies included in this review.

### 3.1. Microbial Diversity in Marine Habitats

Studies focusing on marine snails were quite limited, and we included only one study using conventional techniques [49], with six more using novel molecular techniques [50,51,52,53,54,55] and one using both techniques [56].

**Figure 3:**
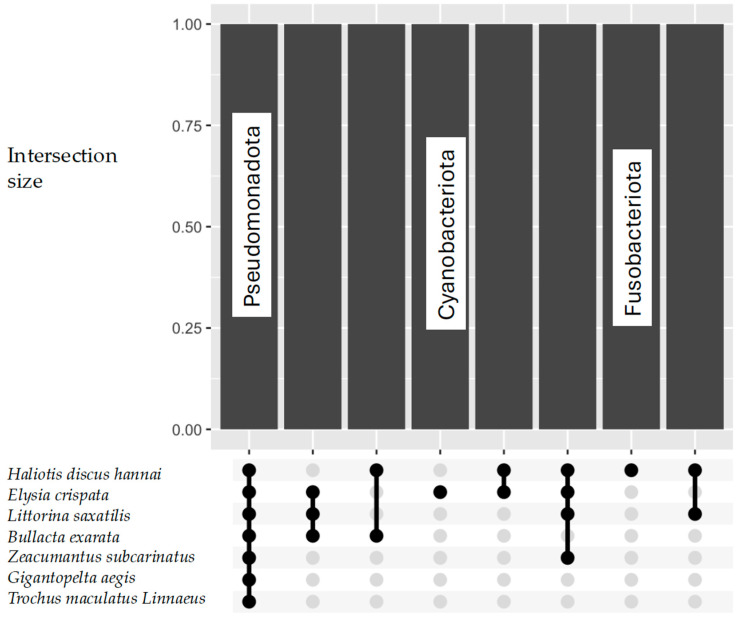
UpsetR plot for Bacteria phyla across marine snail species.

Overall, eight phyla were detected in these studies (Figure 2). Pseudomonadota were the most abundant and the only one detected in all marine snail species (Figure 3). Additionally, Pseudomonadota are known to dominate the microbial communities in various marine habitats, where they represent a significant proportion of the microbial biomass [57,58]. Their high abundance indicates a key role in the microbial dynamics that sustains the health of marine snails by enhancing organic matter decomposition and nutrient availability, while it also implies their potential involvement in digestion and metabolism [59], potentially influencing snails’ growth and reproductive abilities.

Cyanobacteriota, particularly the genus *Synechococcus*, were found only in *Elysia crispata* snails (Figure 3) by [50]. The presence of *Synechococcus* in the diet of gastropods has been linked to enhanced growth and reproductive success, as these cyanobacteria are rich in essential nutrients, which are crucial for the development of these mollusks.

Also, Fusobacteriota (*Psychrilyobacter*) were only mentioned by [53] in *Haliotis discus hannai* snails. *Psychrilyobacter* plays a significant role in the gut microbiome of gastropods, enhancing digestive efficiency [60] and potentially affecting the ecological success of mollusks in various marine environments [61]. Other bacterial phyla occurring in two or three marine snails were Mycoplasmatota, Planctomycetota, and Bacillota.

**Figure 4:**
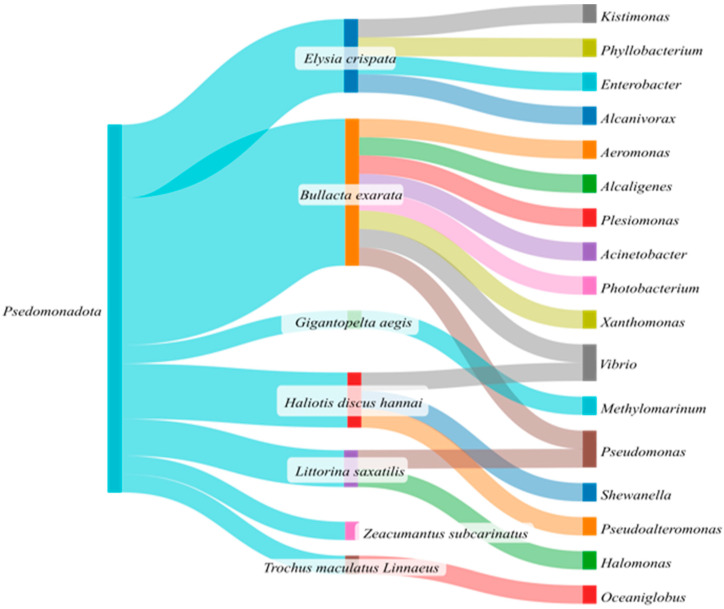
Sankey diagram of the bacterial genera of the Pseudomonadota phylum in marine snails.

Within Pseudomonadota, 17 genera were identified in the reviewed studies (Figure 4). These genera were *Kistimonas*, *Phyllobacterium*, *Enterobacter*, *Alcanivoras*, *Aeromonas*, *Alcaligenes*, *Plesiomonas*, *Acinetobacter*, *Photobacterium*, *Xanthomonas*, *Vibrio*, *Methylomarinum*, *Pseudomonas*, *Shewanella*, *Pseudoalteromonas*, *Halomonas* and *Oceaniglobus*.

*Enterobacter* is potential fermenter [39,62], while *Pseudomonas* is a commonly detected genus in gut samples, mainly known for their proteinolytic activity. These genera of Pseudomonadota bacteria have been recognized for their roles in the gut microbiota of various marine animals. These bacteria play a crucial role in essential metabolic processes, such as nutrient cycling and digestion, which are vital for the health and growth of their hosts. *Aeromonas* and *Vibrio* are frequently found in the gastrointestinal tract of marine organisms, where they can affect gut health and immune functions [63,64]. Additionally, *Pseudoalteromonas* has been identified in the gut microbiota of marine species (sea cucumbers, abalones), indicating its importance in maintaining gut health and potentially providing protection against pathogens [65]. The presence of these bacteria can vary significantly depending on environment and diet, reflecting their adaptability and the dynamic nature of gut microbiota in response to ecological changes [64,66].

*Pseudomonas* and *Vibrio* were the only bacterial genera identified in two different marine snail species, suggesting that these two genera form the bacterial core microbiota of marine snails.

### 3.2. Microbial Diversity in Freshwater Habitats

The bacteria of 22 phyla were collected for this literature review (Figure 2) from studies involving gastropods from freshwater ecosystems [67,68,69,70,71].

Studies focusing on freshwater snails were limited but they have been increased in recent years, showing a trend of increasing interest in them and their microbiota [66,72,73,74]. A total of 21 studies are included in this review, which used conventional [75] or molecular techniques [69,71,76].

**Figure 5:**
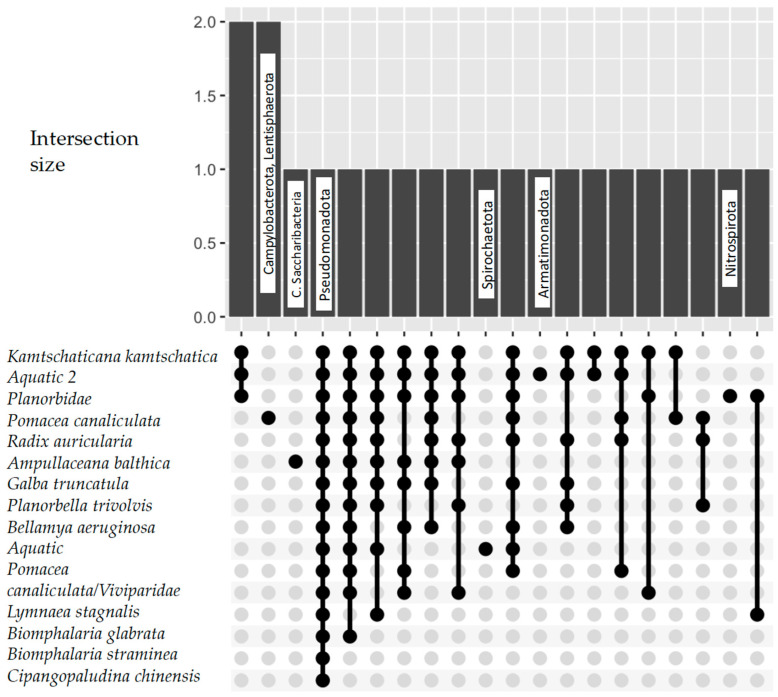
UpsetR plot for Bacteria phyla across freshwater snail species.

Regardless of the sampling site or technique, Pseudomonadota was the dominant phylum in the freshwater gastropods, and they were found in every species (Figure 5). Other abundant phyla were Bacteroidota, Actynomycetota, and Bacillota, which were found to be in common for between 2 and 15 taxa/groups, as identified in the respective papers (Figure 1).

Nitrospirota were found only in snails that were members of the Planorbidae family, while Lentisphaerota and Campylobacterota were identified in *Pomacea caniculata* snails in China. Additionally, *Ampullaceana balthica* was the only species in which *Candidatus Saccharibacteria* was detected. Finally, two studies on freshwater snails (aquatic and aquatic 2; Figure 5) recorded two unique bacteria phyla (Spirochaetota and Armatimonadota).

**Figure 6:**
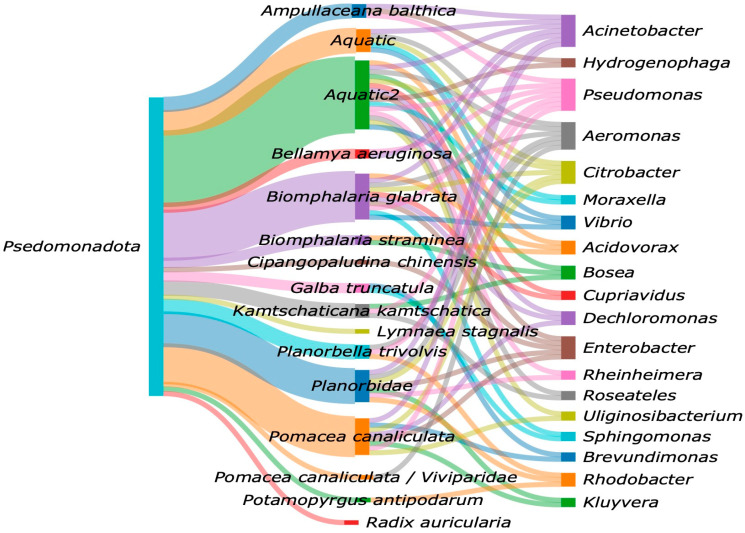
Sankey diagram of the Bacterial genera of Pseudomonadota phylum in freshwater snails.

As shown in Figure 6, at the genus level, we found a dominance of ten Pseudomonadota genera, with *Acinetobacter* and *Pseudomonas* prevailing.

*Enterobacter*, *Pseudomonas*, *Acinetobacter*, *Aeromonas*, and *Vibrio* are also found in marine snails. The prevalence of *Rhodobacter* is of great importance since the genus inhabits different kinds of aquatic environments and possesses the ability to grow both in the light and the dark using photosynthesis, lithotrophy, and both aerobic and anaerobic respiration [77]. *Citrobacter* are potential fermenters [39,62], and it has been recently found that they increase energy harvest in high-fat diets [78]. Also, *Citrobacter* have been previously identified as a cellulotic species [79,80].

The genera *Acidovorax*, *Bosea*, *and Dechloromonas* play significant roles in the gut microbiota of freshwater organisms, contributing to various metabolic processes that are essential for host health. *Acidovorax* have been identified in the gut microbiota of freshwater snails, where they may assist in the degradation of organic compounds thus aiding in nutrient absorption and overall gut function [81]. *Bosea* has been associated with diverse microbial communities in aquatic habitats, indicating its involvement in nutrient cycling, which is crucial for the health of aquatic organisms [82]. *Dechloromonas* have been recognized for their denitrifying capabilities, contributing to nitrogen cycling and good water quality and preventing the accumulation of harmful nitrogenous compounds [83,84]. Regarding the core bacterial taxa, *Pseudomonas* and *Acinetobacter*, *Aeromonas*, *Citrobacter*, and *Enterobacter* were found in at least five snail species in the freshwater habitats noted within this literature review.

### 3.3. Microbial Diversity in Terrestrial Habitats

There were more studies focusing on terrestrial snails as compared to freshwater and marine, totaling 31 papers. A total of 12 studies used molecular techniques [85,86,87,88], 15 studies used conventional techniques [89,90,91,92,93,94,95], and 4 studies used both techniques [96,97,98]. Among all these studies, the majority of them examined bacterial diversity in the intestinal tract and the intestinal extract. However, our review also included studies focusing on crop fluid [91,99].

**Figure 7:**
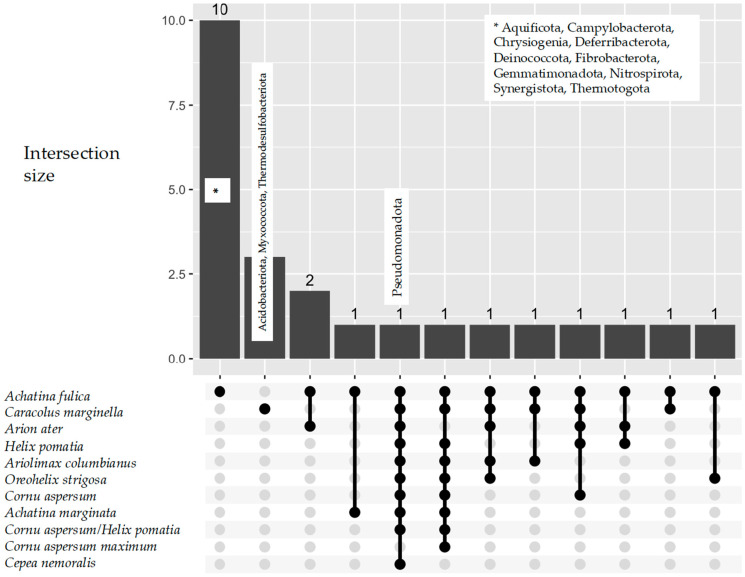
UpsetR plot for Bacteria phyla across terrestrial snail species.

Despite the different molecular and cultural methods used and the different perspectives, all these studies exhibited the prevalence of Pseudomonadota in the gut and other GIT parts of every snail species. The other abundant phylum was the Bacillota, which occurred in eight snail species. Pseudomonadota were not found solely in *Cornu aspersum* maximum (Figure 7); however, in the two studies by [100,101] on this species, the aim was to determine the probiotic properties of *Lactobacillus plantarum* rather than characterize the snail gut microbiota.

The data depict that the following 13 bacteria phyla were unique in two snail species (Figure 7): 3 in *Caracolus marginella* (Thermodesulfobacteriota, Myxococcota, Acidobacteriota) and 10 in *Achatina fulica* (Campylobacterota, Aquificota, Chrysiogenia, Deferribacterota, Deinococcota, Fibrobacterota, Gemmatimonadota, Nitrospirota, Synergistota, Thermotogota).

**Figure 8:**
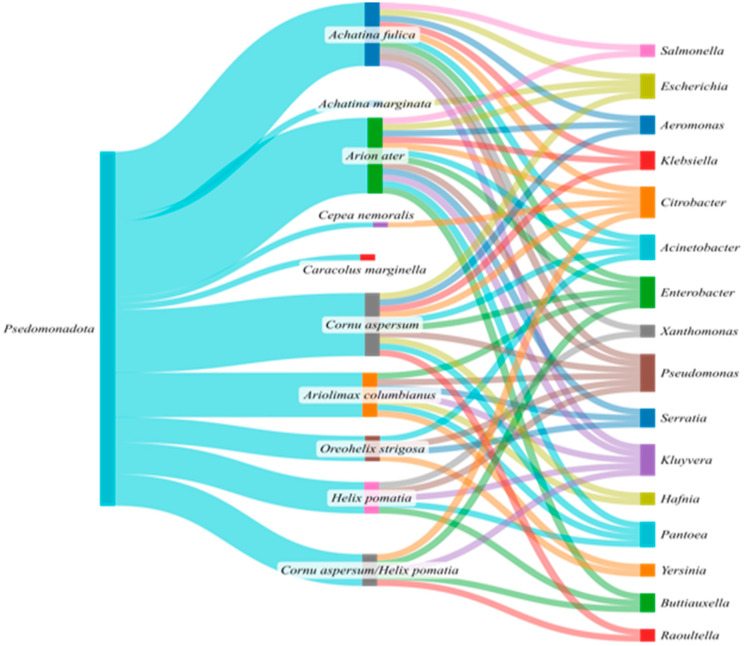
Sankey diagram—Bacteria genus within phylum of Pseudomonadota in terrestrial snails.

Similarly to freshwater and marine snails, the genus that was present in most studies was *Pseudomonas*, which is a facultatively anaerobic fermenter and a potential pathogenic bacterium. Also, as seen in Figure 8, *Enterobacter*, *Citrobacter*, *Escherichia*, *Acinetobacter*, *Kluyvera*, and *Pantoea* prevailed in terrestrial snails. However, other genera such as *Salmonella*, *Raoultella*, *Klebsiella*, *Serratia*, *Hafnia*, *Yersinia*, and *Buttiauxella* were only found in the land snails. *Enterobacter* holds similar metabolic properties and is usually detected in the GIT of several animals. *Acinetobacter*, which has also been associated with marine and freshwater snails [75], has been found in the hemolymph of the Giant African Snail, *Archachatina marginata* [102]. In *A. marginata*, *Acinetobacter* showed cellulase production, indicating a potential role in cellulose digestion [102]. *Citrobacter* and *Kluyvera* were identified by [103] in the intestinal microbiota of terrestrial snails. These bacteria, which are also found in freshwater snails [85,104,105] could play important roles in the digestive process and energy supply of gastropods. *Escherichia coli* can remain in edible land snails, posing public health risks due to the potential transmission to humans [106]. Furthermore, high microbial load levels in farmed snails pose health risks. Research in [90] indicates that *Pantoea and Klebsiella* are prevalent in infected snails, indicating a potential role in disease processes. In addition, the same authors correlated these species occurrence with a decrease in beneficial bacteria (Lactobacillus) in *Cornu aspersum* snails. *Xanthomonas* was the only bacterial genus within Pseudomonadota in land snails [92,97,107] which was also found in marine species [49].

*Pseudomonas*, *Citrobacter*, *Enterobacter*, *Kluyvera*, *Acinetobacter*, *Escherichia*, and *Pantoea* seem to form the core microbiome of terrestrial snails, since they have been identified in at least four different snail species from the reviewed studies. However, the same species that were among the core microbiota in freshwater and marine snails studies were also detected in terrestrial and, more specifically, *Enterobacter* [64,85,87,91,97,105], *Pseudomonas* [49,72,97,105,108,109], *Aeromonas* [66,75,82,105,110], and *Acinetobacter* [49,72,73,85,86,88,99], possibly indicating a core snail gut microbiome, regardless of the habitat. Studies using molecular methods examined GIT bacterial community composition under different aspects including active and estivated animals [88]; lignocellulose metabolism activity [85]; habitat and physiological states [92,111]; age and feed [86]; and host phylogeny [112]. Among the Pseudomonadota, Enterobacterales were usually the most prevalent order [87,90,97,103,105,108,109]. These results and similarities observed with conventional studies further confirm the significance of these bacteria in snail GIT microbial communities. Regarding the functional traits of these bacteria, most of them were shown to possess cellulotic activity. In one study [85] using metagenomic data, it was shown that the glycoside profiles and especially the cellulase, endohemicellulase, and xyloglucanase profiles of slugs and snails were comparable to termites’ profiles for the enzymes responsible for the breakdown/modification of plant cell wall polysaccharides.

Overall, it seems that a significant factor differentiating terrestrial gut microbiome from aquatic gut microbiome is the phylogeny of cellulolytic bacteria. This is most probably due to the different food sources that are available in the land compared to water, implying different kinds of symbiosis. These findings further support the importance of cellulotic bacteria in the GIT of freshwater and terrestrial snails and indicate the importance for their further study for biotechnological applications.

Freshwater species appear to be dominated by taxa belonging to Aeromonadales and Enterobacterales, whereas Vibrionales are predominant in marine species. Also, in terrestrial snails, Enterobacterales and Pseudomonadales are predominant. Ιt is important to note that taxa from Pseudomonadales were found in high abundance in snails from every habitat.

The genomes of the four core genera, i.e., *Acinetobacter*, *Aeromonas*, *Pseudomonas*, and *Enterobacter*, are known to harbor several hydrolases, glycosylases, and glycosidases (Table S1), suggesting the collective cellulolytic potential of ingested plant material by these bacteria in the gut of all snails included in the current review. In addition, earlier studies have noted the importance of the presence of *Aeromonas* in snail gut microbiota, as it may influence their adaptation to new environments and snail health status [66]. Also, *Aeromonas*, *Pseudomonas*, and *Enterobacter* have demonstrated significant cellulolytic activity, playing a crucial role in the degradation of cellulose in the host’s diet [80,97]. Gut microbiota has been profiled in domesticated and farmed animals, including dogs, cats, horses, cows, pigs, and chickens [113,114], but there are even studies on insects such as honeybees [115] and termites [116,117]. Also, many studies on the gut microbiota have been conducted in mammals (wild or captive) [118] and other species used as models in microorganism studies (zebrafish, mice, monkeys) [119,120,121]. Despite the fact that the current coverage of animal-associated microbiomes is quite extensive, snails are not among these animals. Research on land snail microbiomes remains limited, highlighting the need for further studies to understand their ecological and evolutionary significance.

## 4. Conclusions

The current review has shown that the existing literature is far from providing a satisfactory and informative picture of the snails’ microbiomes, despite the lack of obvious reasons for this as there are no major technological limitations for DNA or RNA sequencing approaches. Subsequently, more scientific attention should be paid to investigative approaches in the future. Knowing the keystone core bacterial taxa would allow for the induction of specific studies with these bacteria in order to reveal the mechanisms with which they interact with their hosts. Such studies could include the isolation of snail species-specific strains of these taxa in natural (wild) populations or experimental settings (e.g., nutritional manipulations or pathogen challenges, distinguishing commensals/beneficial with potential pathogens, etc.). Whether in natural or controlled settings, nucleic acid-based studies, namely metagenomics and metatranscriptomics, can be complemented with proteomics and/or metabolomics to investigate the chemical crosstalk between bacterial symbionts and their hosts. Of course, the expansion of the snails’ microbial diversity seems imperative as these animals live in habitats with highly diverse and variable microbial populations and have complex—but in some cases, well-described—life cycles. Finally, the relatively easy and cost-effective way to maintain snail population under laboratory conditions could render them an appropriate non-model animal system for various microbiome studies, such as symbionts acquisitions and heritability.

We expect that this literature review will serve as a starting point on the current state-of-the-art studies so that the relevant researchers will prioritize the most promising directions towards understanding and exploiting snail microbiomes. Snail microbiota and microbiomes can provide a new insight into the ecology, ecophysiology, evolution, and conservation of wild and farmed terrestrial and aquatic gastropods as holobionts [122].

## Supplementary Materials

The following supporting information can be downloaded at: https://www.mdpi.com/article/10.3390/microorganisms13010008/s1, List of papers used for this review S1; Table S1: Table of hydrolases, glycosylases, and glycosidases harbor the genomes of the four core genera. Refs. [123,124,125,126,127,128,129,130,131,132,133,134,135,136,137,138,139,140,141,142,143,144,145,146,147,148,149,150,151,152,153,154,155,156,157,158,159,160,161,162,163,164,165,166,167,168,169,170,171,172,173,174,175,176,177,178,179,180,181,182,183,184,185,186,187,188,189,190,191,192,193,194,195,196,197,198,199,200,201,202,203,204,205,206,207] can be found in Supplementary Materials.
