# Supplementary material for: Bacterial Diversity Associated with Terrestrial and Aquatic Snails
Source: Microorganisms. 2024 Dec 24;13(1):8. doi: 10.3390/microorganisms13010008 (PMC11767905; doi:10.3390/microorganisms13010008)
Supplement: Supplementary file 1 [file microorganisms-13-00008-s001.zip › microorganisms-3351428-supplementary.pdf]

List of papers used for this review S1

1. Abdraba, A.M.; Saleuddin, A.S. Localization and Immunological Characterization of Insulin-like Peptide(s) in the Land Snail *Otala lactea* (Mollusca: Pulmonata). *Can. J. Zool.* **2000**, *78*, 1515–1526, doi:10.1139/z00-052.
2. Aceves, A.K.; Johnson, P.D.; Atkinson, C.L.; Van Ee, B.C.; Bullard, S.A.; Arias, C.R. Digestive Gland Microbiome of *Pleurobema cordatum*: Mesocosms Induce Dysbiosis. *Journal of Molluscan Studies* **2020**, *86*, 280–289, doi:10.1093/mollus/eyaa024.
3. Ademolu, K.O., Ojo, V.O., Bamidele, J.A., Adelabu, A.B., Ebenso, I., Idowu, A.B.. Feeding pattern and gut enzymes activity of Giant African land snail (*Archachatina marginata*) during growth phases. Archivos de Zootecnia **2017**, 66 (253), pp. 29-34.
4. Adeyeye, E.I. Waste Yield, Proximate and Mineral Composition of Three Different Types of Land Snails Found in Nigeria. *International Journal of Food Sciences and Nutrition* **1996**, *47*, 111–116, doi:10.3109/09637489609012572.
5. Aksenov, A.S.; Kisil, O.Ya.; Chervochkina, A.S.; Khrebtova, I.S.; Mantsurova, K.S.; Bespalaya, Yu.V.; Aksenova, O.V. Bacterial Communities within the Freshwater Lymnaeid Snail *Kamtschaticana Kamtschatica* (Middendorff, 1850) in Northeastern Siberia. *Microbiology* **2024**, *93*, 180–183, doi:10.1134/S0026261723603883.
6. Ansart, A., Nicolai, A., Vernon, P., Madec, L. Do ice nucleating agents limit the supercooling ability of the land snail Cornu aspersum? Cryo-Letters **2010**, 31 (4), pp. 329-340.
7. Ansart, A.; Vernon, P.; Charrier, M.; Daguzan, J. The Effect of Antibiotic Treatment on the Supercooling Ability of the Land Snail *Helix Aspersa* (Gastropoda: Pulmonata). *Cryobiology* **2002**, *44*, 189–192, doi:10.1016/S0011-2240(02)00015-9.
8. Ariole, C.; Asuzu, C. THE EFFECT OF CRUDE OIL ON INTESTINAL MICROBIAL POPULATIONS OF A FRESHWATER SNAIL. *J. Glob. Biosci.* **2013**, *2*, 132–138.
9. Aronson, H.S.; Zellmer, A.J.; Goffredi, S.K. The Specific and Exclusive Microbiome of the Deep-Sea Bone-Eating Snail, *Rubyspira Osteovora*. *FEMS Microbiol. Ecol.* **2017**, *93*, fiw250, doi:10.1093/femsec/fiw250.
10. Awharitoma, A.O.; Okaka, C.E.; Obaze, S.E. Larval Stages of Brachylaima Fuscatum in the Terrestrial Snail *Limicolaria Aurora* from Southern Nigeria. *Journal of Helminthology* **2003**, *77*, 1–5, doi:10.1079/JOH2002155.
11. Barber, A.; Dean, J.; Jordana, R.; Ponz, F. Sugar and Amino Acid Intestinal Transport Systems in Land Snail *Helix Aspersa*. *Revista Espanola de Fisiologia* **1989**, *45*, 215–224.
12. Bayne, C.J. Molluscan Internal Defense Mechanism: The Fate of C14-Labelled Bacteria in the Land snail *Helix Pomatia* (L.). *J. Comp. Physiol.* **1973**, *86*, 17–25, doi:10.1007/BF00694474.
13. Bayne, C.J. Molluscan Immunity: Induction of Elevated Immunity in the Land Snail *(Helix)* by Injections of Bacteria (*Pseudomonas Aeruginosa*). *Developmental & Comparative Immunology* **1980**, *4*, 43–54, doi:10.1016/S0145-305X(80)80007-3.
14. Bertani, R.; Cosimi, C.; De Liso, A.; Ferrara, R.; Maserti, B.E.; Trifoglio, M.; Zuccarelli, D. Mercury in a Primary Consumer (*Eobania Vermiculata*) Collected near a Chlor-alkali Complex. *Environmental Technology* **1994**, *15*, 1095–1100, doi:10.1080/09593339409385519.
15. Bluem, V.; Paris, F. Aquatic Modules for Bioregenerative Life Support Systems Based on the C.E.B.A.S. Biotechnology. *Acta Astronautica* **2001**, *48*, 287–297, doi:10.1016/S0094-5765(01)00025-X.
16. Bluem, V.; Paris, F. Novel Aquatic Modules for Bioregenerative Life-Support Systems Based on the Closed Equilibrated Biological Aquatic System (c.e.b.a.s.). *Acta Astronautica* **2002**, *50*, 775–785, doi:10.1016/S0094-5765(02)00014-0.
17. Boomer, S.M.; Baltzley, M.J.; Dutton, B.E.; Smith, P.N. The Faecal Microbiome of the Pacific Banana Slug, *Ariolimax Columbianus*, Displays Seasonal Variation. *J. Molluscan Stud.* **2024**, *90*, eyae007, doi:10.1093/mollus/eyae007.

18. Boyer, S.; Wratten, S.D.; Holyoake, A.; Abdelkrim, J.; Cruickshank, R.H. Using Next-Generation Sequencing to Analyse the Diet of a Highly Endangered Land Snail (Powelliphanta Augusta) Feeding on Endemic Earthworms. *PLOS ONE* **2013**, *8*, e75962, doi:10.1371/journal.pone.0075962.
19. Boyer, S.; Yeates, G.W.; Wratten, S.D.; Holyoake, A.; Cruickshank, R.H. Molecular and Morphological Analyses of Faeces to Investigate the Diet of Earthworm Predators: Example of a Carnivorous Land Snail Endemic to New Zealand. *Pedobiologia* **2011**, *54*, S153–S158, doi:10.1016/j.pedobi.2011.08.002.
20. Brown, P. Our Crowded Niche. *Natural History* **2004**, *113*, 8–9.
21. Butcher, A.R.; Grove, D.I. Description of the Life-Cycle Stages of Brachylaima Cribbi n. Sp. (Digenea: Brachylaimidae) Derived from Eggs Recovered from Human Faeces in Australia. *Syst Parasitol* **2001**, *49*, 211–221, doi:10.1023/A:1010616920412.
22. Butina, T.V.; Zemskaya, T.I.; Bondaryuk, A.N.; Petrushin, I.S.; Khanaev, I.V.; Nebesnykh, I.A.; Bukin, Y.S. Viral Diversity in Samples of Freshwater Gastropods Benedictia Baicalensis (Caenogastropoda: Benedictiidae) Revealed by Total RNA-Sequencing. *International Journal of Molecular Sciences* **2023**, *24*, 17022, doi:10.3390/ijms242317022.
23. Cardoso, A.M., Cavalcante, J.J.V., Cantao, M.E., Thompson, C.E., Flatschart, R.B., Glogauer, A., Scapin, S.M.N., Sade, Y.B., Beltrao, P.J.M.S.I., Gerber, A.L., Martins, O.B., Garcia, E.S., de Souza, W., Vasconcelos, A.T.R. Metagenomic Analysis of the Microbiota from the Crop of an Invasive Snail Reveals a Rich Reservoir of Novel Genes. PLoS ONE **2012**, 7 (11), art. no. e48505.
24. Cardoso, A.M., Cavalcante, J.J.V., Vieira, R.P., Lima, J.L., Grieco, M.A.B., Clementino, M.M., Vasconcelos, A.T.R., Garcia, E.S., de Souza, W., Albano, R.M., Martins, O.B. Gut bacterial communities in the giant land snail *Achatina fulica* and their modification by sugarcane-based diet. PLoS ONE **2012**, 7 (3), art. no. e33440.
25. Caullan, L.P.; Vila, G.G.; Angulo, E.A.; Calvo, A.; Marcelo, J.A.; Torras, M.A.C. Microbiota from *Helix aspersa* Müller in Barcelona Area (Spain). *Adv. Microbiol.* **2014**, *2014*, doi:10.4236/aim.2014.410066.
26. Chalifour, B.N.; Elder, L.E.; Li, J. Gut Microbiome of Century-Old Snail Specimens Stable across Time in Preservation. *Microbiome* **2022**, *10*, 99, doi:10.1186/s40168-022-01286-z.
27. Chalifour, B.; Li, J. Characterization of the Gut Microbiome in Wild Rocky Mountain snails (*Oreohelix Strigosa*). *Anim. Microbiome* **2021**, *3*, 49, doi:10.1186/s42523-021-00111-6.
28. Charrier, M., Combet-Blanc, Y., Ollivier, B. Bacterial flora in the gut of *Helix aspersa* (Gastropoda Pulmonota): evidence for a permanent population with a dominant homolactic intestinal bacterium, *Enterococcus casseliflavus*. Canadian Journal of Microbiology **1998**, 44: 20-27.
29. Charrier, M.Y.; Fonty, G.; Gaillard-Martinie, B.; Ainouche, K.; Andant, G. Isolation and Characterization of Cultivable Fermentative Bacteria from the Intestine of Two Edible Snails, *Helix pomatia and Cornu aspersum* (Gastropoda: Pulmonata). *Biol. Res.* **2006**, *39*, doi:10.4067/S0716-97602006000500010.
30. Chen, L.; Li, S.; Xiao, Q.; Lin, Y.; Li, X.; Qu, Y.; Wu, G.; Li, H. Composition and Diversity of Gut Microbiota in *Pomacea canaliculata* in Sexes and between Developmental Stages. *BMC Microbiol.* **2021**, *21*, 200, doi:10.1186/s12866-021-02259-2.
31. Chen, Z.; Yun, S.-T.; Zhang, M.-J.; Rui Yin; Zhou, Y.-X. *Oceaniglobus Trochenteri Sp.* Nov., Isolated from the Gut Microflora of Top Shell (*Trochus maculatus Linnaeus*). *Arch. Microbiol.* **2021**, *203*, 5613–5619, doi:10.1007/s00203-021-02543-9.
32. Christensen, S.C.B.; Nissen, E.; Arvin, E.; Albrechtsen, H.-J. Distribution of *Asellus aquaticus* and Microinvertebrates in a Non-Chlorinated Drinking Water Supply System – Effects of Pipe Material and Sedimentation. *Water Research* **2011**, *45*, 3215–3224, doi:10.1016/j.watres.2011.03.039.
33. Coulis, M.; Hättenschwiler, S.; Rapior, S.; Coq, S. The Fate of Condensed Tannins during Litter Consumption by Soil Animals. *Soil Biology and Biochemistry* **2009**, *41*, 2573–2578, doi:10.1016/j.soilbio.2009.09.022.

34. Dallinger, R.; Wieser, W. Patterns of Accumulation, Distribution and Liberation of Zn, Cu, Cd and Pb in Different Organs of the Land Snail *Helix pomatia* L. *Comparative Biochemistry and Physiology Part C: Comparative Pharmacology* **1984**, *79*, 117–124, doi:10.1016/0742-8413(84)90173-7.
35. Dang, V.T.; Speck, P.; Doroudi, M.; Smith, B.; Benkendorff, K. Variation in the Antiviral and Antibacterial Activity of Abalone *Haliotis laevigata*, *H. rubra* and Their Hybrid in South Australia. *Aquaculture* **2011**, *315*, 242–249, doi:10.1016/j.aquaculture.2011.03.005.
36. Dar, M.A.; Pawar, K.D.; Jadhav, J.P.; Pandit, R.S. Isolation of Cellulolytic Bacteria from the Gastro-Intestinal Tract of *Achatina Fulica* (Gastropoda: Pulmonata) and Their Evaluation for Cellulose Biodegradation. *Int. Biodeterior. Biodegrad.* **2015**, *98*, 73–80, doi:10.1016/j.ibiod.2014.11.016.
37. Dar, M.A.; Pawar, K.D.; Pandit, R.S. Prospecting the Gut Fluid of Giant African Land Snail, *Achatina Fulica* for Cellulose Degrading Bacteria. *Int. Biodeterior. Biodegrad.* **2018**, *126*, 103–111, doi:10.1016/j.ibiod.2017.10.006.
38. Davidson, G.L.; Cienfuegos, I.A.; Dalesman, S. Antibiotic-Altered Gut Microbiota Explain Host Memory Plasticity and Disrupt Pace-of-Life Covariation for an Aquatic Snail. *The ISME Journal* **2024**, *18*, wrae078, doi:10.1093/ismejo/wrae078.
39. Deidei, H.; Amadi, L.; Aleruchi, O. Impact of Various Slime Removal Treatment and Antibiogram of Bacterial Isolates from Snails (*Achatina fulica*) Harvested in Port Harcourt Metropolis. **2024**.
40. Dullo, W.-C.; Gektidis, M.; Golubic, S.; Heiss, G.A.; Kampmann, H.; Kiene, W.; Kroll, D.K.; Kuhrau, M.L.; Radtke, G.; Reijmer, J.G.; et al. Factors Controlling Holocene Reef Growth: An Interdisciplinary Approach. *Facies* **1995**, *32*, 145–188, doi:10.1007/BF02536867.
41. Dushku, E.; Ioannou, A.; Staikou, A.; Yiangou, M. Probiotic Properties and Immunomodulatory Activity of Gastrointestinal Tract Commensal Bacterial Strains Isolated from the Edible Farmed Snail *Cornu aspersum* Maxima. *Fish Shellfish Immunol.* **2019**, *92*, 792–801, doi: 10.1016/j.fsi.2019.06.061.
42. Dushku, E.; Kotzamanidis, C.; Avgousti, K.; Zdragas, A.; Vafeas, G.; Giantzi, V.; Staikou, A.; Yiangou, M. *Listeria monocytogenes* Induced Dysbiosis in Snails and Rebiosis Achieved by Administration of the Gut Commensal *Lactobacillus plantarum* Sgs14 Strain. *Fish Shellfish Immunol.* **2020**, *104*, 337–346, doi: 10.1016/j.fsi.2020.04.041.
43. EFFORD, M. Consumption of amphipods by the New Zealand land snail *Wainuia urnula* (Pulmonata: Rhytididae). *Journal of Molluscan Studies* **2000**, *66*, 45–52, doi:10.1093/mollus/66.1.45.
44. Efstratiou, E.; Feidantsis, K.; Makri, V.; Staikou, A.; Giantsis, I.A. Evidence for Beneficial Physiological Responses of the Land Snail *Cornu aspersum* to Probiotics' (*Lactobacillus plantarum*) Dietary Intervention. *Animals* **2024**, *14*, 857, doi:10.3390/ani14060857.
45. El-Samanody, E.-S.A.; AbouEl-Enein, S.A.; Emara, E.M. Molecular Modeling, Spectral Investigation and Thermal Studies of the New Asymmetric Schiff Base Ligand; (E)-N′-(1-(4-((E)-2-Hydroxybenzylideneamino) Phenyl) Ethylidene)Morpholine-4-Carbothiohydrazide and Its Metal Complexes: Evaluation of Their Antibacterial and Anti-Molluscicidal Activity. *Applied Organometallic Chemistry* **2018**, *32*, e4262, doi:10.1002/aoc.4262. 1.
46. Errani, F.; Ciulli, S.; Mandrioli, L.; Serratore, P.; Volpe, E. Detection of Human and Fish Viruses in Marine Gastropods. *Animals* **2022**, *12*, 2122, doi:10.3390/ani12162122.
47. Février, Y.; Russo, J.; Madec, L. Intraspecific Variation in Life History Traits of a Land Snail after a Bacterial Challenge. *Journal of Zoology* **2009**, *277*, 149–156, doi:10.1111/j.1469-7998.2008.00523.x.
48. Gállego, L.; Gracenea, M. Effect of Praziquantel on the Tegument and Digestive Epithelium Ultrastructure of Brachylaima Sp. Metacercariae Parasitizing the Edible Land Snail *Cornu aspersum*. *Journal of Parasitology* **2016**, *102*, 520–532, doi:10.1645/16-65.

49. Ghose, K.C. Observations on the digestive enzymes and cellulolytic bacteria of the giant land snail *Achatina fulica* and their occurrence in the gastropoda. Proceedings of the Zoological Society of London, **1961,** 137 (1), pp. 127-133.
50. Cloherty, T.M.; Rachlin, J.W. Physicochemical and Shoreline Development Factors Affecting Lake Littoral Benthic Macroinvertebrates. *Journal of Freshwater Ecology* **2011**, *26*, 517–525, doi:10.1080/02705060.2011.588435.
51. Gracenea, M.; Gállego, L. Brachylaimiasis: Brachylaima Spp. (Digenea: Brachylaimidae) Metacercariae Parasitizing the Edible Snail *Cornu aspersum* (Helicidae) in Spanish Public Marketplaces and Health-Associated Risk Factors. *Journal of Parasitology* **2017**, *103*, 440–450, doi:10.1645/17-29.
52. Guoliang, W.; Tianlun, Z.; Tongxia, L.; Yinong, W.; Hong, Y.; Shan, J. Bacteriological Analysis of the Digestive Tube of the Mud Snail (Bullacta exarata Philippi) and Its Rearing Shoal. *J. Ocean Univ. Qingdao* **2002**, *1*, 161–164, doi:10.1007/s11802-002-0012-x.
53. Harris, J.M. The Presence, Nature, and Role of Gut Microflora in Aquatic Invertebrates: A Synthesis. *Microb. Ecol.* **1993**, *25*, 195–231, doi:10.1007/BF00171889.
54. Haskins, N.; Panglao, M.; Qu, Q.; Majumdar, H.; Cabrera-Luque, J.; Morizono, H.; Tuchman, M.; Caldovic, L. Inversion of Allosteric Effect of Arginine on N-Acetylglutamate Synthase, a Molecular Marker for Evolution of Tetrapods. *BMC Biochem* **2008**, *9*, 24, doi:10.1186/1471-2091-9-24.
55. Henchiri, M. Depositional Morphotypes and Implications of the Quaternary Travertine and Tufa Deposits from along Gafsa Fault: Jebel El Mida, Southwestern Tunisia. *Journal of African Earth Sciences* **2014**, *90*, 9–24, doi:10.1016/j.jafrearsci.2013.10.012.
56. Herlemann, D.P.R.; Tammert, H.; Kivistik, C.; Käiro, K.; Kisand, V. Distinct Biogeographical Patterns in Snail Gastrointestinal Tract Bacterial Communities Compared with Sediment and Water. *MicrobiologyOpen* **2024**, *13*, e13, doi:10.1002/mbo3.1413.
57. Hong, S.; Khim, J.S.; Park, J.; Son, H.-S.; Choi, S.-D.; Choi, K.; Ryu, J.; Kim, C.-Y.; Chang, G.S.; Giesy, J.P. Species- and Tissue-Specific Bioaccumulation of Arsenicals in Various Aquatic Organisms from a Highly Industrialized Area in the Pohang City, Korea. *Environmental Pollution* **2014**, *192*, 27–35, doi:10.1016/j.envpol.2014.05.004.
58. Hu, Z.; Chang, J.; Tong, Q.; Yu, J.; Li, S.; Niu, H. [High-throughput sequencing analysis of intestinal flora diversity of two freshwater snails (*Radix auricularia and Planorbella trivolvis*)]. *Sheng Wu Gong Cheng Xue Bao Chin. J. Biotechnol.* **2020**, *36*, 2622–2634, doi:10.13345/j.cjb.200322.
59. Hu, Z.; Chen, X.; Chang, J.; Yu, J.; Tong, Q.; Li, S.; Niu, H. Compositional and Predicted Functional Analysis of the Gut Microbiota of *Radix auricularia* (Linnaeus) via High-Throughput Illumina Sequencing. *PeerJ* **2018**, *6*, e5537, doi:10.7717/peerj.5537.
60. Hur, S.-W.; Cadangin, J.; Lee, S.; Lee, J.-H.; Park, S.-J.; Jang, W.-J.; Choi, Y.-H. Dietary Replacement of *Undaria Pinnatifida* by *Sargassum Horneri* in Feed Formulation for *Abalone Haliotis discus hannai*: Effect on Growth, Gut Microbiota, and Taste Sensory Profile. *Front. Mar. Sci.* **2023**, *10*, doi:10.3389/fmars.2023.1053240.
61. Ivanova, E.S.; Spiridonov, S.E.; Clark, W.C.; Tourna, M.; Wilson, M.J.; Barker, G.M. Description and Systematic Affinity of *Alaninema ngata* n. Sp. (Alaninematidae: Panagrolaimorpha) Parasitising Leaf-Veined Slugs (Athoracophoridae: Pulmonata) in New Zealand. **2013**, doi:10.1163/15685411-00002724.
62. Joynson, R.; Pritchard, L.; Osemwekha, E.; Ferry, N. Metagenomic Analysis of the Gut Microbiome of the Common Black Slug *Arion ater* in Search of Novel Lignocellulose Degrading Enzymes. *Front. Microbiol.* **2017**, *8*, 2181, doi:10.3389/fmicb.2017.02181.
63. Joynson, R.; Swamy, A.; Bou, P.A.; Chapuis, A.; Ferry, N. Characterization of Cellulolytic Activity in the Gut of the Terrestrial Land Slug *Arion Ater*: Biochemical Identification of Targets for Intensive Study. *Comp. Biochem. Physiol. B Biochem. Mol. Biol.* **2014**, *177–178*, 29–35, doi:10.1016/j.cbpb.2014.08.003.

64. Kelly, B.; Mtiti, E.; McIntyre, P.B.; Vadeboncoeur, Y. Stable Isotopes Reveal Nitrogen Loading to Lake Tanganyika from Remote Shoreline Villages. *Environmental Management* **2017**, *59*, 264–273, doi:10.1007/s00267-016-0787-y. 1.
65. Kivistik, C.; Tammert, H.; Kisand, V.; Käiro, K.; Herlemann, D.P.R. Impact of Disturbance and Dietary Shift on Gastrointestinal Bacterial Community and Its Invertebrate Host System. *Mol. Ecol.* **2023**, *32*, 6631–6643, doi:10.1111/mec.16628.
66. Koch, E., Lozada M., Dionisi H., Castro-Vazquez A. Uric acid-degrading bacteria in the gut of the invading apple snail *Pomacea canaliculata* and their possible symbiotic significance. Symbiosis, **2014**, 63:149–155.
67. Koleva, Z.; Dedov, I.; Kizheva, J.; Lipovanska, R.; Moncheva, P.; Hristova, P. Lactic Acid Microflora of the Gut of Snail *Cornu aspersum*. *Biotechnol. Biotechnol. Equip.* **2014**, *28*, 627–634, doi:10.1080/13102818.2014.947071
68. Koleva, Z.V.; Kizheva, Y.K.; Tishkov, S.H.; Dedov, I.K.; Kirova, E.L.; Stefanova, P.M.; Moncheva, P.A.; Hristova, P.K. Dynamics of Bacterial Community in the Gut of Cornu Aspersum. *J. Biosci. Biotechnol.* **2015**, *4*.
69. Kotzamanidis, C.; Malousi, A.; Dushku, E.; Dobly, A.; De Keersmaecker, S.C.J.; Roosens, N.H.; Karathodorou, A.; Staikou, A.; Zdragas, A.; Yiangou, M. *Listeria monocytogenes* Isolates from *Cornu aspersum* Snails: Whole Genome-Based Characterization and Host-Pathogen Interactions in a Snail Infection Model. *Fish & Shellfish Immunology* **2022**, *123*, 469–478, doi:10.1016/j.fsi.2022.03.028.
70. Kowalczyk-Pecka, D.; Puchalski, A. Potential Interaction between the *Cepaea nemoralis* Wild Snail and Citrobacter Spp. Bacteria. **2008**.
71. Kunselman, E.; Wiggin, K.; Diner, R.E.; Gilbert, J.A.; Allard, S.M. Microbial Threats and Sustainable Solutions for Molluscan Aquaculture. *Sustainable Microbiology* **2024**, *1*, qvae002, doi:10.1093/sumbio/qvae002.
72. Lan, Y.; Sun, J.; Chen, C.; Sun, Y.; Zhou, Y.; Yang, Y.; Zhang, W.; Li, R.; Zhou, K.; Wong, W.C.; et al. Hologenome Analysis Reveals Dual Symbiosis in the Deep-Sea Hydrothermal Vent Snail *Gigantopelta aegis*. *Nat. Commun.* **2021**, *12*, 1165, doi:10.1038/s41467-021-21450-7.
73. Langford, G.J.; Willobee, B.A.; Isidoro, L.F. Transmission, Host Specificity, and Seasonal Occurrence of *Cyrtosomum penneri* (Nematoda: Atractidae) in Lizards from Florida. *Journal of Parasitology* **2013**, *99*, 241–246, doi:10.1645/12-30.1.
74. Li, P.; Hong, J.; Wu, M.; Yuan, Z.; Li, D.; Wu, Z.; Sun, X.; Lin, D. Metagenomic Analysis Reveals Variations in Gut Microbiomes of the Schistosoma Mansoni-Transmitting Snails *Biomphalaria straminea and Biomphalaria glabrata*. *Microorganisms* **2023**, *11*, 2419, doi:10.3390/microorganisms11102419.
75. Li, P.; Hong, J.; Yuan, Z.; Huang, Y.; Wu, M.; Ding, T.; Wu, Z.; Sun, X.; Lin, D. Gut Microbiota in Parasite-Transmitting Gastropods. *Infect. Dis. Poverty* **2023**, *12*, 105, doi:10.1186/s40249-023-01159-z.
76. Li L.-H., Lv S., Lu Y., Bi D.-Q., Guo Y.-H., Wu J.-T., Yue Z.-Y., Mao G.-Y., Guo Z.-X., Zhang Y., Tang Y.-F. Spatial structure of the microbiome in the gut of *Pomacea canaliculata*. BMC Microbiology, **2019,** 19:273
77. Lin, D.; Hong, J.; Sanogo, B.; Du, S.; Xiang, S.; Hui, J.H.-L.; Ding, T.; Wu, Z.; Sun, X. Core Gut Microbes *Cloacibacterium and Aeromonas* Associated with Different Gastropod Species Could Be Persistently Transmitted across Multiple Generations. *Microbiome* **2023**, *11*, 267, doi:10.1186/s40168-023-01700-0
78. Lin, Z.; Torres, J.P.; Ammon, M.A.; Marett, L.; Teichert, R.W.; Reilly, C.A.; Kwan, J.C.; Hughen, R.W.; Flores, M.; Tianero, M.D.; et al. A Bacterial Source for Mollusk Pyrone Polyketides. *Cell Chemical Biology* **2013**, *20*, 73–81, doi:10.1016/j.chembiol.2012.10.019.
79. Liu, H.; Yang, X.; Yang, W.; Zheng, Z.; Zhu, J. Gut Microbiota of Freshwater Gastropod (*Bellamya aeruginosa*) Assist the Adaptation of Host to Toxic Cyanobacterial Stress. *Toxins* **2023**, *15*, 252, doi:10.3390/toxins15040252.

80. Luizaga, L.R., da Silva, F.L., Silva, L.B. Microbiological conditions and occurrence of the African land snail in Araguaina city at Tocantins State, Brazil. JOURNAL OF BIOENERGY AND FOOD SCIENCE, **2015**, 2(4):234-238. 1.
81. Mahadevan, P.; Middlebrooks, M.L. Bacterial Diversity in the Clarki Ecotype of the Photosynthetic Sacoglossan, *Elysia crispata*. *MicrobiologyOpen* **2020**, *9*, e1098, doi:10.1002/mbo3.1098.
82. Martinez-Pereira, M.A.; Franceschi, R. da C.; Antunes, G. de F.; Coelho, B.P.; Achaval, M.; Zancan, D.M. General Morphology and Innervation of the Midgut and Hindgut of *Megalobulimus abbreviatus* (Gastropoda, Pulmonata). *jzoo* **2013**, *30*, 319–330, doi:10.2108/zsj.30.319.
83. Mazzillo, F.F.M.; Shapiro, K.; Silver, M.W. A New Pathogen Transmission Mechanism in the Ocean: The Case of Sea Otter Exposure to the Land-Parasite *Toxoplasma gondii*. *PLOS ONE* **2013**, *8*, e82477, doi:10.1371/journal.pone.0082477.
84. McCann, P.; McFarland, C.; Megaw, J.; Siu-Ting, K.; Cantacessi, C.; Rinaldi, G.; Gobert, G.N. Assessing the Microbiota of the Snail Intermediate Host of Trematodes, *Galba truncatula*. *Parasit. Vectors* **2024**, *17*, 31, doi:10.1186/s13071-024-06118-7.
85. Michaelidis, B.; Hatzikamari, M.; Antoniou, V.; Anestis, A.; Lazou, A. Stress Activated Protein Kinases, JNKs and P38 MAPK, Are Differentially Activated in Ganglia and Heart of Land Snail *Helix lucorum* (L.) during Seasonal Hibernation and Arousal. *Comparative Biochemistry and Physiology Part A: Molecular & Integrative Physiology* **2009**, *153*, 149–153, doi:10.1016/j.cbpa.2009.01.021.
86. Munn, A.J.; Treloar, M. Phenotypic Plasticity in the Common Garden Snail: Big Guts and Heavier Mucus Glands Compete in Snails Faced with the Dual Challenge of Poor Diet and Coarse Substrate. *J Comp Physiol B* **2017**, *187*, 545–561, doi:10.1007/s00360-016-1051-8.
87. Nakao, M.; Waki, T.; Sasaki, M.; Anders, J.L.; Koga, D.; Asakawa, M. *Brachylaima Ezohelicis* Sp. Nov. (Trematoda: Brachylaimidae) Found from the Land Snail *Ezohelix gainesi*, with a Note of an Unidentified *Brachylaima* Species in Hokkaido, Japan. *Parasitology International* **2017**, *66*, 240–249, doi:10.1016/j.parint.2017.01.015.
88. Naya, D.E.; Catalán, T.; Artacho, P.; Gaitán-Espitia, J.D.; Nespolo, R.F. Exploring the Functional Association between Physiological Plasticity, Climatic Variability, and Geographical Latitude: Lessons from Land Snails. *Evol Ecol Res* **2011**, *13*, 647–659.
89. Nicolai, A.; Rouland-Lefèvre, C.; Ansart, A.; Filser, J.; Lenz, R.; Pando, A.; Charrier, M. Inter-Population Differences and Seasonal Dynamic of the Bacterial Gut Community in the Endangered Land Snail *Helix pomatia* (Gastropoda: Helicidae). *Malacologia* **2015**, *59*, 177–190, doi:10.4002/040.059.0101.
90. Nicolai, A.; Vernon, P.; Lee, M.; Ansart, A.; Charrier, M. Supercooling Ability in Two Populations of the Land Snail *Helix pomatia* (Gastropoda: Helicidae) and Ice-Nucleating Activity of Gut Bacteria. *Cryobiology* **2005**, *50*, 48–57, doi:10.1016/j.cryobiol.2004.10.003.
91. Niu, Y.; Cao, W.; Zhao, Y.; Zhai, H.; Zhao, Y.; Tang, X.; Chen, Q. The Levels of Oxidative Stress and Antioxidant Capacity in Hibernating *Nanorana Parkeri*. *Comparative Biochemistry and Physiology Part A: Molecular & Integrative Physiology* **2018**, *219–220*, 19–27, doi:10.1016/j.cbpa.2018.02.003.
92. Ngumah, C.; Ogbulie, J.; Orji, J.; Amadi, E.; Nweke, C.; Allino, J. Optimizing Biomethanation of a Lignocellulosic Biomass Using Indigenous Microbial-Cellulases Systems. *BioTechnologia. Journal of Biotechnology Computational Biology and Bionanotechnology* **2017**, *98*. 1.
93. North, E.; Minton, R.L. Diversity and Predicted Function of Gut Microbes from Two Species of Viviparid Snails. *Freshw. Mollusk Biol. Conserv.* **2021**, *24*, 104–113, doi:10.31931/fmbc-d-20-00008.
94. Núñez C.G., Torras M.A.C. Comparison of the Microbiota of Snails (*Helix aspersa*) of Different Weights and Its Evolution over Time. Journal of Veterinary Science & Technology, **2015**, 6:216. 1.

95. Ojewole, J.A. Studies on the Responses of Isolated Intestine of the Giant African Land Snail (*Archachatina marginata*) to Drugs. *Biochem Exp Biol* **1980**, *16*, 31–37.
96. Olivares-Rubio, H.F.; Salazar-Coria, L.; Vega-López, A.; Olivares-Rubio, H.F.; Salazar-Coria, L.; Vega-López, A. Oxidative Stress, Lipid Metabolism, and Neurotransmission in Freshwater Snail (*Pomacea patula*) Exposed to a Water-Accommodated Fraction of Crude Oil. *Hidrobiológica* **2017**, *27*, 265–280.
97. Panova, M.A.Z.; Varfolomeeva, M.A.; Gafarova, E.R.; Maltseva, A.L.; Mikhailova, N.A.; Granovitch, A.I. First Insights into the Gut Microbiomes and the Diet of the *Littorina* Snail Ecotypes, a Recently Emerged Marine Evolutionary Model. *Evol. Appl.* **2023**, *16*, 365–378, doi:10.1111/eva.13447.
98. Parvate, Y.A.; Liji Thayil, L.T. Toxic Effect of Clove Oil on the Survival and Histology of Various Tissues of Pestiferous Land Snail *Achatina fulica* (Bowdich, 1822). **2017**. 1.
99. Parkyn, S.M.; Collier, K.J.; Hicks, B.J. New Zealand Stream Crayfish: Functional Omnivores but Trophic Predators? *Freshwater Biology* **2001**, *46*, 641–652, doi:10.1046/j.1365-2427.2001.00702.x.
100. Pawar, K.D.; Banskar, S.; Rane, S.D.; Charan, S.S.; Kulkarni, G.J.; Sawant, S.S.; Ghate, H.V.; Patole, M.S.; Shouche, Y.S. Bacterial Diversity in Different Regions of Gastrointestinal Tract of Giant African Snail (Chatina Fulica). *Microbiology Open* **2012**, *1*, 415–426, doi:10.1002/mbo3.38.
101. Pawar, K.D.; Dar, M.A.; Rajput, B.P.; Kulkarni, G.J. Enrichment and Identification of Cellulolytic Bacteria from the Gastrointestinal Tract of Giant African Snail, *Achatina fulica*. *Appl. Biochem. Biotechnol.* **2015**, *175*, 1971–1980, doi:10.1007/s12010-014-1379-z.
102. Petrella, B.; Goudsmit, E.M.; Ketchum, P.A. Isolation of a Galactogen Utilizing Strain of Arthrobacter. *Arch. Microbiol.* **1980**, *124*, 87–89, doi:10.1007/BF00407033.
103. Pinheiro, G.L.; Correa, R.; Soares, R.; Cardoso, A.; Chaia, C.; Clementino, M.M.; Garcia, E.; De Souza, W.; Frases, S. Isolation of Aerobic Cultivable Cellulolytic Bacteria from Different Regions of the Gastrointestinal Tract of Giant Land Snail Achatina Fulica. *Front. Microbiol.* **2015**, *6*, doi:10.3389/fmicb.2015.00860.
104. Pinheiro, G.L.; Rodriguez, J.E.; Domont, G.B.; de Souza, W.; Junqueira, M.; Frases, S. Biochemical Characterization of Streptomyces Sp. I1.2 Secretome Reveals the Presence of Multienzymatic Complexes Containing Cellulases and Accessory Enzymes. *BioEnergy Res.* **2017**, *10*, 1–12, doi:10.1007/s12155-016-9771-x.
105. Pokora, Z. [Role of gastropods in epidemiology of human parasitic diseases]. *Wiad Parazytol* **2001**, *47*, 3–24.
106. Ponce, J.J.; Arismendi, I.; Thomas, A. Using In-Situ Environmental DNA Sampling to Detect the Invasive New Zealand Mud Snail (*Potamopyrgus antipodarum*) in Freshwaters. *PeerJ* **2021**, *9*, e11835, doi:10.7717/peerj.11835.
107. Preu, P.; Braun, M. German SIMBOX on Chinese Mission Shenzhou-8: Europe's First Bilateral Cooperation Utilizing China's Shenzhou Programme. *Acta Astronautica* **2014**, *94*, 584–591, doi:10.1016/j.actaastro.2013.08.022.
108. Rabelo-Fernandez, R.J.; Santiago-Morales, K.; Morales-Vale, L.; Rios-Velazquez, C. The Metagenome of *Caracolus marginella* Gut Microbiome Using Culture Independent Approaches and Shotgun Sequencing. *Data Brief* **2018**, *16*, 501–505, doi:10.1016/j.dib.2017.11.043.1.
109. Redlarski, G.; Lewczuk, B.; Żak, A.; Koncicki, A.; Krawczuk, M.; Piechocki, J.; Jakubiuk, K.; Tojza, P.; Jaworski, J.; Ambroziak, D.; et al. The Influence of Electromagnetic Pollution on Living Organisms: Historical Trends and Forecasting Changes. *BioMed Research International* **2015**, *2015*, 234098, doi:10.1155/2015/234098.
110. Richardson, A.M.M. Food, Feeding Rates and Assimilation in the Land Snail *Cepaea nemoralis* L. *Oecologia* **1975**, *19*, 59–70, doi:10.1007/BF00377590.
111. Riddle, W.A. Cold Hardines in the Woodland Snail, *Anguispira alternata* (Say) (Endodontidae). *Journal of Thermal Biology* **1981**, *6*, 117–120, doi:10.1016/0306-4565(81)90046-2.

112. Röszer, T.; Kiss-Tóth, É.; József Szentmiklósi, A.; Bánfalvi, G. Seasonal Periodicity of Enteric Nitric Oxide Synthesis and Its Regulation in the Snail, Helix Lucorum. *Invertebrate Biology* **2005**, *124*, 18–24, doi:10.1111/j.1744-7410.2005.1241-03.x.
113. Russell, L.K.; DeHaven, J.I.; Botts, R.P. Toxic Effects of Cadmium on the Garden Snail (*Helix aspersa*). *Bull. Environ. Contam. Toxicol.; (United States)* **1981**, *26:5*, doi:10.1007/BF01622148.
114. Russo, J.; Madec, L. Dual Strategy for Immune Defense in the Land Snail *Cornu aspersum* (Gastropoda, Pulmonata). *Physiological and Biochemical Zoology* **2011**, *84*, 212–221, doi:10.1086/659123.
115. Russo, J.; Madec, L. Linking Immune Patterns and Life History Shows Two Distinct Defense Strategies in Land Snails (Gastropoda, Pulmonata). *Physiological and Biochemical Zoology* **2013**, *86*, 193–204, doi:10.1086/669482.
116. Salloum, P.M.; Jorge, F.; Poulin, R. Different Trematode Parasites in the Same Snail Host: Species-specific or Shared Microbiota? *Mol. Ecol.* **2023**, *32*, 5414–5428, doi:10.1111/mec.17111.
117. Schoettli, G.; Seiler, H.G. Uptake and Localization of Radioactive Zinc in the Visceral Complex of the Land pulmonate *Arion rufus*. *Experientia* **1970**, *26*, 1212–1213, doi:10.1007/BF01897971.
118. Schols, R.; Vanoverberghe, I.; Huyse, T.; Decaestecker, E. Host-Bacteriome Transplants of the Schistosome Snail Host *Biomphalaria glabrata* Reflect Species-Specific Associations. *FEMS Microbiology Ecology* **2023**, *99*, fiad101, doi:10.1093/femsec/fiad101.
119. Shi, Z.; Yao, F.; Chen, Q.; Chen, Y.; Zhang, J.; Guo, J.; Zhang, S.; Zhang, C. More Deterministic Assembly Constrains the Diversity of Gut Microbiota in Freshwater Snails. *Front. Microbiol.* **2024**, *15*, doi:10.3389/fmicb.2024.1394463.
120. Schweiger, F.; Kuhn, M. *Dicrocoelium dendriticum* Infection in a Patient with Crohn's Disease. *Canadian Journal of Gastroenterology and Hepatology* **2008**, *22*, 912791, doi:10.1155/2008/912791.
121. Silva, T.M.; Melo, E.S.; Lopes, A.C.S.; Veras, D.L.; Duarte, C.R.; Alves, L.C.; Brayner, F.A. Characterization of the Bacterial Microbiota of *Biomphalaria Glabrata* (Say, 1818) (Mollusca: Gastropoda) from Brazil. *Lett. Appl. Microbiol.* **2013**, *57*, 19–25, doi:10.1111/lam.12068.
122. Sun, X.; Hong, J.; Ding, T.; Wu, Z.; Lin, D. Snail Microbiota and Snail–Schistosome Interactions: Axenic and Gnotobiotic Technologies. *Trends Parasitol.* **2024**, *40*, 241–256, doi:10.1016/j.pt.2024.01.002.
123. Tada, S.; Hori, M.; Yamaoka, K.; Hata, H. Diversification of Functional Morphology in Herbivorous Cichlids (Perciformes: Cichlidae) of the Tribe Tropheini in Lake Tanganyika. *Hydrobiologia* **2017**, *791*, 83–101, doi:10.1007/s10750-016-2761-3.
124. Takacs-Vesbach, C.; King, K.; Van Horn, D.; Larkin, K.; Neiman, M. Distinct Bacterial Microbiomes in Sexual and Asexual *Potamopyrgus antipodarum*, a New Zealand Freshwater Snail. *PLOS ONE* **2016**, *11*, e0161050, doi:10.1371/journal.pone.0161050.
125. Tramell, P.R.; Campbell, J.W. Carbamyl Phosphate Synthesis in Invertebrates. *Comparative Biochemistry and Physiology Part B: Comparative Biochemistry* **1971**, *40*, 395–406, doi:10.1016/0305-0491(71)90224-0.
126. Van Horn, D.J.; Garcia, J.R.; Loker, E.S.; Mitchell, K.R.; Mkoji, G.M.; Adema, C.M.; Takacs-Vesbach, C.D. Complex Intestinal Bacterial Communities in Three Species of Planorbid Snails. *J. Molluscan Stud.* **2012**, *78*, 74–80, doi:10.1093/mollus/eyr038.
127. Villena M.A., Morales S.C., Soto J.O., Enciso M.H. Bacterial flora in the digestive tract of *Helix aspersa* Müller snails under two breeding systems. Revista Investigaciones Veterinarias del Perú, **2010**, 21(1): 100-105
128. Wada, S.; Kawakami, K.; Chiba, S. Snails Can Survive Passage through a Bird's Digestive System. *Journal of Biogeography* **2012**, *39*, 69–73, doi:10.1111/j.1365-2699.2011.02559.x.
129. Waterhouse, B.R.; Boyer, S.; Wratten, S.D. Pyrosequencing of Prey DNA in Faeces of Carnivorous Land Snails to Facilitate Ecological Restoration and Relocation Programmes. *Oecologia* **2014**, *175*, 737–746, doi:10.1007/s00442-014-2933-7.
130. Woodman, H.E. The Role of Cellulose in Nutrition. *Biological Reviews* **1930**, *5*, 273–295, doi:10.1111/j.1469-185X.1930.tb00900.x.

131. Weiner, S.; Noff, D.; Meyer, M.S.; Weisman, Y.; Edelstein, S. Metabolism of Cholecalciferol in Land Snails. *Biochemical Journal* **1979**, *184*, 157–161, doi:10.1042/bj1840157.
132. Xiang, X.; Chen, M.; Wu, C.; Zhu, A.; Yang, J.; Lv, Z.; Wang, T. Glycolytic Regulation in Aestivation of the Sea Cucumber Apostichopus Japonicus: Evidence from Metabolite Quantification and Rate-Limiting Enzyme Analyses. *Mar Biol* **2016**, *163*, 167, doi:10.1007/s00227-016-2936-5.
133. Zaady, E.; Offer, Z.Y.; Shachak, M. The Content and Contributions of Deposited Aeolian Organic Matter in a Dry Land Ecosystem of the Negev Desert, Israel. *Atmospheric Environment* **2001**, *35*, 769–776, doi:10.1016/S1352-2310(00)00263-6.
134. Zhang, T.; Zhu, H.; Wang, J.; Lin, X.; Wang, J.; Huang, Y.; Li, B.; Mou, H.; Ma, X.; Wang, R. Monitoring Bacterial Community Dynamics in Abalone (*Haliotis discus Hannai*) and the Correlations Associated with Aquatic Diseases. *Water* **2022**, *14*, 1769, doi:10.3390/w14111769.
135. Zhao, Y.; Yang, H.; Storey, K.B.; Chen, M. RNA-Seq Dependent Transcriptional Analysis Unveils Gene Expression Profile in the Intestine of Sea Cucumber *Apostichopus Japonicus* during Aestivation. *Comparative Biochemistry and Physiology Part D: Genomics and Proteomics* **2014**, *10*, 30–43, doi:10.1016/j.cbd.2014.02.002.
136. Zhong, J.; Wang, W.; Yang, X.; Yan, X.; Liu, R. A Novel Cysteine-Rich Antimicrobial Peptide from the Mucus of the Snail of *Achatina fulica*. *Peptides* **2013**, *39*, 1–5, doi:10.1016/j.peptides.2012.09.001.
137. Zhou, Z.; Wu, H.; Li, D.; Zeng, W.; Huang, J.; Wu, Z. Comparison of Gut Microbiome in the Chinese Mud Snail (*Cipangopaludina chinensis*) and the Invasive Golden Apple Snail (*Pomacea canaliculata). PeerJ* **2022**, *10*, e13245, doi:10.7717/peerj.13245.

Table S1: Table of hydrolases, glycosylases, and glycosidases harbor the genomes of the four core genera.

Bacteria	Type of Cellulase	Enzyme	Procedure	KEGG nomenclature
		alpha-L-rhamnosidase; alpha-L-rhamnosidase T;alpha-L-rhamnosidase N	Hydrolysis of terminal non-reducing alpha-L-rhamnose residues in alpha-L-rhamnosides	EC 3.2.1.40
		pullulanase;<br>limit dextrinase (erroneous);<br>amylopectin 6-glucanohydrolase;<br>bacterial debranching enzyme;<br>debranching enzyme;<br>alpha-dextrin endo-1,6-alpha-glucosidase;<br>R-enzyme;<br>pullulan alpha-1,6-glucanohydrolase	Hydrolysis of (1->6)-alpha-D-glucosidic linkages in pullulan, amylopectin and glycogen, and in the alpha- and beta-limit dextrins of amylopectin and glycogen	EC 3.2.1.41
Core (Enterobacter, Acinetobacter, Pseudomonas, Aeromonas)	Endocellulases	glucosylceramidase;<br>psychosine hydrolase;<br>glucosphingosine glucosylhydrolase;<br>GlcCer-beta-glucosidase;<br>beta-D-glucocerebrosidase;<br>glucosylcerebrosidase;<br>beta-glucosylceramidase;<br>ceramide glucosidase;<br>glucocerebrosidase;<br>glucosylsphingosine beta-glucosidase;<br>glucosylsphingosine beta-D-glucosidase		EC 3.2.1.45
		galactosylceramidase;<br>cerebroside galactosidase;<br>galactocerebroside.beta-galactosidase;<br>galactosylcerebrosidase;<br>galactocerebrosidase;<br>ceramide galactosidase;<br>galactocerebroside galactosidase;<br>galactosylceramide.beta-galactosidase;<br>cerebroside beta-galactosidase;<br>galactosylceramidase I;<br>beta-galactosylceramidase;<br>galactocerebroside-beta-D-galactosidase;<br>lactosylceramidase I;	a D-galactosyl-N-acylsphingosine + H_2O = D-galactose + a ceramide	EC 3.2.1.46

	beta-galactocerebrosidase; lactosylceramidase		
	sucrose alpha-glucosidase; sucrose alpha-glucohydrolase; sucrase; sucrase-isomaltase; sucrose.alpha.-glucohydrolase; intestinal sucrase; sucrase(invertase)	Hydrolysis of sucrose and maltose by an alpha-D-glucosidase-type action	EC 3.2.1.48
	alpha-N-acetylgalactosaminidase; alpha-acetylgalactosaminidase; N-acetyl-alpha-D-galactosaminidase; N-acetyl-alpha-galactosaminidase; alpha-NAGAL; alpha-NAGA; alpha-GalNAcase	Cleavage of non-reducing alpha-(1->3)-N-acetylgalactosamine residues from human blood group A and AB mucin glycoproteins, Forssman hapten and blood group A lacto series glycolipids	EC 3.2.1.49
Exocellulases	cellulose 1,4-beta-cellobiosidase (non-reducing end); exo-cellobiohydrolase; beta-1,4-glucan cellobiohydrolase; beta-1,4-glucan cellobiosylhydrolase; 1,4-beta-glucan cellobiosidase; exoglucanase; avicelase; CBH 1; C1 cellulase; cellobiohydrolase I; cellobiohydrolase; exo-beta-1,4-glucan cellobiohydrolase; 1,4-beta-D-glucan cellobiohydrolase; cellobiosidase	Hydrolysis of (1->4)-beta-D-glucosidic linkages in cellulose and cellotetraose, releasing cellobiose from the non-reducing ends of the chains	EC 3.2.1.91
β-glucosidases	beta-glucosidase; gentiobiase; cellobiase; emulsin; elaterase; aryl-beta-glucosidase; beta-D-glucosidase; beta-glucoside glucohydrolase; arbutinase; amygdalinase; p-nitrophenyl beta-glucosidase; primeverosidase;	Hydrolysis of terminal, non-reducing beta-D-glucosyl residues with release of beta-D-glucose	EC 3.2.1.21

	amygdalase; linamarase; salicilinase; beta-1,6-glucosidase		
	endoplasmic reticulum Man8GlcNAc2 1,2-alpha-mannosidase; MNL1	Man8GlcNAc2-[protein] (isomer 8A1,2,3B1,3) + H2O = Man7GlcNAc2-[protein] (isomer 7A1,2,3B3) + D-mannopyranose	EC 3.2.1.210
	galactan exo-1,6-beta-galactobiohydrolase (non-reducing end); exo-beta-1,6-galactobiohydrolase; 1,6Gal	Hydrolysis of (1->6)-beta-D-galactosidic linkages in arabinogalactan proteins and (1->3):(1->6)-beta-galactans to yield (1->6)-beta-galactobiose as the final product	EC 3.2.1.213
	exo beta-1,2-glucooligosaccharide sophorohydrolase	[(1->2)-beta-D-glucosyl]n + H2O = sophorose + [(1->2)-beta-D-glucosyl]n-2	EC 3.2.1.214
	kojibiose hydrolase; kojibiase	kojibiose + H2O = beta-D-glucopyranose + D-glucopyranose	EC 3.2.1.216
	exo-acting protein-alpha-N-acetylgalactosaminidase; Nag31	a [protein]-N-acetyl-alpha-D-galactosalaminyl-(L-serine/L-threonine) + H2O = a [protein]-(L-serine/L-threonine) + N-acetyl-D-galactosamine	EC 3.2.1.217
	alpha-3'-ketoglucosidase; 3'-keto-alpha-D-gluco-disaccharide hydrolase; alpha-3-ketoglucosidase (incorrect); 3-keto-glucoside hydrolase	3'-dehydrosucrose + H2O = 3-dehydro-D-glucopyranose + D-fructofuranose	EC 3.2.1.218
	palatinase; palQ	palatinose + H2O = alpha-D-glucopyranose + D-fructofuranose	EC 3.2.1.219
